# A Synthetic, Small, Sulfated Agent Is a Promising Inhibitor of *Chlamydia spp*. Infection *in vivo*

**DOI:** 10.3389/fmicb.2018.03269

**Published:** 2019-01-16

**Authors:** Karen M. Gallegos, Christopher R. Taylor, Daniel J. Rabulinski, Rosalinda Del Toro, Danielle E. Girgis, Dapinder Jourha, Vaibhav Tiwari, Umesh R. Desai, Kyle H. Ramsey

**Affiliations:** ^1^Department of Microbiology and Immunology, College of Graduate Studies, Midwestern University, Downers Grove, IL, United States; ^2^Department of Dermatology, Charles C. Gates Center for Regenerative Medicine and Stem Cell Biology, University of Colorado – Anschutz Medical Campus, Aurora, CO, United States; ^3^Institute for Structural Biology, Drug Discovery and Development, Department of Medicinal Chemistry, Virginia Commonwealth University, Richmond, VA, United States

**Keywords:** *Chlamydia trachomatis*, *Chlamydia muridarum*, inhibition, glucoside, SPGG

## Abstract

*Chlamydia* is the most frequently reported sexually transmitted bacteria causing 2.9 million infections annually in the United States. Diagnosis, treatment, and sequelae of chlamydial disease cost billions of dollars each year in the United States alone. Considering that a heparin sulfate-like cell surface receptor is involved in *Chlamydia* infections, we reasoned that sulfated and sulfonated mimics of heparin sulfate would be useful in topical prophylactic prevention of *Chlamydia*. In this study, we tested a small, synthetic sulfated agent sulfated pentagalloyl glucoside (SPGG) and three synthetic sulfonated polymers PSS and SPS with average molecular weight in the range of 11 to 1000 kDa for inhibition against *Chlamydia*. Infection of HeLa cells with *C. muridarum* or *C. trachomatis* in the presence of increasing concentrations of SPGG or sulfonated polymers were quantified by immunofluorescence of *Chlamydia* inclusions. To determine whether *in vitro* pre-treatment of SPGG inhibits infection of *C. muridarum*, HeLa monolayers were incubated with SPGG-containing media, and then infected with *Chlamydia*. Our *in vitro* results show that SPGG pre-treatment inhibits *Chlamydia* infection in a dose-dependent manner. In addition, we further determined if SPGG treatment has an inhibitory effect during infection, therefore cell monolayers were infected with *C. muridarum* in the concurrent presence of SPGG. Our results show that SPGG inhibits *C. muridarum* infection with an IC_50_ at 10 μg/ml levels. We also tested the inhibitory effect of synthetic polymers PSS and SPS against *Chlamydia* and found inhibition of *C. muridarum* and *C. trachomatis* infections with IC_50_ ranging from 0.3 to 0.8 μg/ml. SPGG, PSS, and SPS inhibit formation of *Chlamydia* inclusions in a concentration-dependent manner. For evaluation of *in vivo* efficacy of the most effective agent in blocking *C. muridarum*, SPGG, we intravaginally pre-treated mice with SPGG before infection with *C. muridarum*. Cervical swabs were collected post-infection to quantify *Chlamydia* inclusions *in vitro*. Our *in vivo* data show that the SPGG-treated group has a statistically significant reduction of infection compared to the no-treatment control. Overall, our results show that SPGG could serve as a promising topical inhibitor for preventing *Chlamydia* infection.

## Introduction

*Chlamydia sp*. are obligate intracellular pathogens and the most common sexually transmitted bacteria worldwide (World Health Organization [WHO], 2016). Human *Chlamydia spp.* cause several diseases such as cervicitis, trachoma, urethritis, ectopic pregnancy, pelvic inflammatory disease, lymphogranuloma venerum (LGV) as well as others (Häcker, 2018). *Chlamydia trachomatis* infection is the leading cause of blindness worldwide, while *C. muridarum*, the murine pathogen closely related to *C. trachomatis*, is commonly used for modeling human *Chlamydial* infections in mouse.

Annually, about 20 million people acquire sexually transmitted infections (STI) in the United States. Moreover, in 2016 alone, about 1.5 million cases of *C. trachomatis* infection were reported to the Centers of Disease Control (Centers for Disease Control and Prevention [CDC], 2017). Although recommended antibiotic treatment for *Chlamydia* infections is generally effective and antibiotic resistance is thus far rare (Sandoz and Rockey, 2010), most of the infected patients are unlikely to seek treatment. This is because 70 to 90% of *Chlamydia* infections in women and >50% in men are asymptomatic (Centers for Disease Control and Prevention [CDC], 2017). Untreated *Chlamydia* infections often cause serious sequelae and complications that lead to infertility (Haggerty et al., 2010; Darville, 2013; Centers for Disease Control and Prevention [CDC], 2017). Furthermore, it has been reported that the standard antibiotic treatment against *Chlamydia* is less effective than expected possibly due to persistent infections (Kissinger et al., 2016). Although several efforts have been made in public health programs to improve screening and medical treatment to control *Chlamydia*, the incidence of *Chlamydia* infection has increased (Rekart and Brunham, 2008; World Health Organization Library [WHO], 2012).

Numerous approaches have been proposed to address this problem; one of which is the use of topical microbicidal or microbistatic prophylaxis. It is believed that new intravaginal compounds that prevent adherence or growth of STI could be effective (Achilles et al., 2002; Stone, 2002; Tiwari et al., 2012; Osaka and Hefty, 2014). Earlier work has shown that heparan sulfate (HS)-like cell surface receptors are involved in mediating *Chlamydia* infections (Wuppermann et al., 2001; Rosmarin et al., 2012; Tiwari et al., 2012), which has led to the use of new sulfated and sulfonated agents as broad-spectrum inhibitors of STI (Herold et al., 1997, 2000; Simoes et al., 2002; Scordi-Bello et al., 2005). Inhibition of *Chlamydia spp.* infection (*C. trachomatis* serovar LGV, *C. muridarum* serovars Nigg) was achieved by exogenous heparin and other negatively charged agents along with cell lines defective in the synthesis of HS (Zaretzky et al., 1995; Wuppermann et al., 2001; Bourne et al., 2003). Although some *C. trachomatis* serovars (e.g., E and D) were shown to have an infectivity mechanism independent of HS (Taraktchoglou et al., 2001), pretreatment with negatively charged agents are also reported to inhibit these serovars (Zaretzky et al., 1995; Herold et al., 1997, 2000; Achilles et al., 2002).Therefore, we reasoned that agents carrying optimal sulfate or sulfonate groups would potently block microbial attachment and/or reduce host cell-pathogen interaction, thereby reducing *Chlamydia* infections. This in turn could help reduce transmission of infection and need for antibiotics, possibly reducing incidences of the sequelae of *Chlamydia* infections.

In this study, we evaluated the anti-*Chlamydia* activities of a synthetic, small, sulfated agent called sulfated pentagalloyl glucoside (SPGG) with average molecular weight (MW) of 2.2 kDa (structure shown in Supplemental Figure S1), synthetic polymers called poly(sodium 4-styrene sulfonate; PSS) with average MW of 1000 kDa, poly(4-styrenesulfonic acid; PSS) with average MW of 75 kDa, and another synthetic polymer called polyanetholsulfonic acid sodium salt (SPS) with average MW of 11 kDa. The objective of this study is to test the inhibitory activity of sulfated and sulfonated compounds against *Chlamydia*. We evaluated the ability of these four different agents to (1) block interaction of host cell with *Chlamydia* and (2) inhibit *Chlamydia* growth.

## Materials and Methods

### Mammalian Cell Lines, *Chlamydia* Stocks, and Reagents

HeLa 229 cells (epithelial cervix adenocarcinoma, ATCC CCL-2.1) were maintained in DMEM media supplemented with 5% heat-inactivated fetal bovine serum and 1 μg/ml gentamicin at 37°C 5% CO_2_. For these studies, we evaluated *C. muridarum* Nigg and Weiss strains, as well as *C. trachomatis* serovar LGV. We obtained all *Chlamydia* strains from Midwestern University *Chlamydia* stocks. Initially, *C. muridarum* Weiss strain was obtained from Dr. T. Cotter who acquired the stocks from laboratory of Dr. H. Caldwell. Dr. H. Caldwell originally obtained this Weiss isolate from J. Schachter, who had obtained it from E. Weiss (Swenson et al., 1983; Ramsey et al., 2009). *C. muridarum* Nigg strain was originally obtained from Dr. R. Rank of the University of Arkansas who obtained from American Type Culture Collection (Rockville, MD, United States) (Hilleman, 1945). SPGG with average MW of 2.2 kDa was synthesized in the Desai laboratory at Virginia Commonwealth University following earlier reports (Al-Horani et al., 2013; Al-Horani and Desai, 2014). PSS with MWs of 75 and 1000 kDa and SPS with a MW of 11 kDa were obtained from Sigma-Aldrich (Catalog #: 561223, 434574, and 444464, respectively). All reagents were used as received, dissolved or diluted in Milli-Q water for further evaluation. Reagent stocks were prepared at high concentrations (50–100 mg/ml) to minimize any effect of the drug diluent (Milli-Q water) after dilution to the working concentrations tested in this study (ng/ml–μg/ml). Therefore, untreated control replicates were only treated with media only and diluent was not included in any of these replicates.

### Cytotoxicity Assay

To evaluate the cytotoxicity of the compounds used in this study, we determined the viability of tissue culture cells by 3-(4,5-dimethylthiazol-2-yl)-2,5-diphenyltetrazolium bromide (MTT) assay in a 96 well plate. HeLa 229 cells were treated for 24 h in a humidified 5% CO_2_ incubator at 37°C with different concentrations of the compounds. After incubation, 10 μL of MTT stock solution (5 mg/ml) was added to adherent cells and incubated for four additional hours. Acidic isopropanol (0.1 N HCl in absolute isopropanol) was added after the incubation and absorbance of converted dye was measured at 570 nm with background subtraction at 630 nm.

### *Chlamydia in vitro* Drug Activity Assays

For pretreatment of monolayer assays, confluent HeLa 229 cell monolayers were drug treated or untreated for 30 min at 37°C, 5% CO_2_ with different drug concentrations. After the drug treatment, drug containing media was removed and suspension of *Chlamydia* elementary bodies (EBs) was used to inoculate monolayers in 24-well microtiter plates as described previously (Cotter et al., 1997). Inoculum was rocked for 1 h at room temperature. Plates were then incubated for two additional hours at 37°C, 5% CO_2_. After the incubation period, fresh media containing 1 μg/ml of cycloheximide was added. *C. muridarum* assays were incubated 24 h while *C. trachomatis* assays were incubated for 48 h at 37°C, 5% CO_2_ before plates were fixed with methanol. *C. muridarum* inclusions were visualized by staining with the α-*Chlamydia* mouse anti-serum paired with secondary FITC-anti mouse antibody (Thermo Fisher Scientific, IL, United States) while *C. trachomatis* inclusions were stained with commercial biotin-labeled polyclonal α-*Chlamydia* antibody (Fitzgerald Industries International, MA, United States) paired with FITC streptavidin (Invitrogen Corporation, CA, United States). *Chlamydia* growth was determined by counting fluorescent inclusions viewed through an inverted fluorescence microscope. For pre-treatment of EBs assays, untreated cell monolayers were inoculated with drug treated EBs. *Chlamydia* EBs were drug treated or untreated for 30 min at room temperature, centrifuged for 45 min at 25300 *g*, drug containing media was removed, and EBs were resuspended in drug-free media before using them as inoculum. For co-treatment, *Chlamydia* EB inoculum was added simultaneously with drug containing media onto monolayers. Both were rocked for 1 h at room temperature and then incubated for 2 h at 37°C, 5% CO_2_. After the incubation time, plates were processed as indicated above. *Chlamydia* infectivity was calculated by counting the inclusion-forming units (IFU) using a Zeiss Axiovert 25 fluorescent microscope to calculate the IFU/ml of each sample. A minimum of 20 fields were counted for each replicate; a minimum of two replicates were evaluated per treatment. The IFU/ml was calculated by multiplying the total number of inclusions obtained per sample by the dilution factor of the sample and by the field factor and divining the final number by the volume of the sample. The field factors vary on the number of fields counted, the area of the wells in each plate and the magnification used. For example for these studies, the field factor for a 24-well plate (Genesee Scientific Corporation, CA, United States) with 20 counted fields at magnification of 40×, has a field factor of 37.26.

### Animal Studies

Female outbred Swiss Webster mice (35–42 days old and about 20 g in body weight) were obtained from Charles River Laboratories and used for all experiments. All mice were acclimated to the Animal Resource Facility for at least 7 days prior to inclusion in the experiments. The animal experiments had cohorts that received SPGG topically at different concentrations; in addition, untreated control groups or no-drug treated groups were included. All mice had free access to food and water during the course of these studies and were maintained on a 10:14 (light: dark) cycle. All animal experimental protocols were approved by the Institutional Animal Care and Use Committee. Using a mouse *Chlamydia* infection model previously described (Ramsey et al., 2001), all mice were treated subcutaneously with 100 μL of phosphate-buffered saline (PBS, 0.14 M NaCl, 0.01 M phosphate buffer, pH 7.4) containing 2.5 mg of medroxyprogesterone acetate (Greenstone LLC, NJ, United States) 7 days prior to intravaginal infection. The animal study comprised cohorts that received 30-min topical intravaginal treatment: one cohort received PBS buffer only, one cohort received vehicle gel only and three cohorts received increasing concentrations of SPGG in vehicle gel before *Chlamydia* infection. The vehicle gel was prepared with 2% hydroxyethylcellulose (HEC) in PBS. On the day of infection, after the 30-min treatment, all cohorts were inoculated intravaginally with 10 μl of *C. muridarum* Weiss inoculum containing 10^6^ IFU in Sucrose - Phosphate - Glutamate (SPG) buffer (10 mM phosphate, 0.25 M sucrose, 5 mM L-glutamic acid, pH 7.4). To evaluate *Chlamydia* infection from the lower urogenital tract, cervicovaginal swabs with polyester tipped applicators (Puritan^®^medical products, ME, United States) were collected. Samples were collected at 4, 7, 10, 14, and 21 days post-infection. All samples were frozen in SPG buffer at –80°C for later batch processing. *Chlamydia* inclusions were quantified from swabs samples in HeLa 229 cell cultures and enumerated by indirect immunofluorescence as previously described (Cotter et al., 1997).

### Statistical Analysis

*Chlamydia* infectivity was calculated by normalizing raw values of inclusion number to percent of the untreated control group or negative control value. In addition, the areas under the treatment time curve (AUC) were calculated for the *in vivo* experiment data using GraphPad Prism 7 (La Jolla, CA, United States). To determine the effect of the compounds, the concentration of compound where the inhibition is reduced by half (IC_50_) or in 90% (IC_90_) were determined and different therapies were compared using Student’s *t*-test analysis using GraphPad Prism 7 software.

## Results

### Activity of Compounds Against *Chlamydia*

**FIGURE 1:**
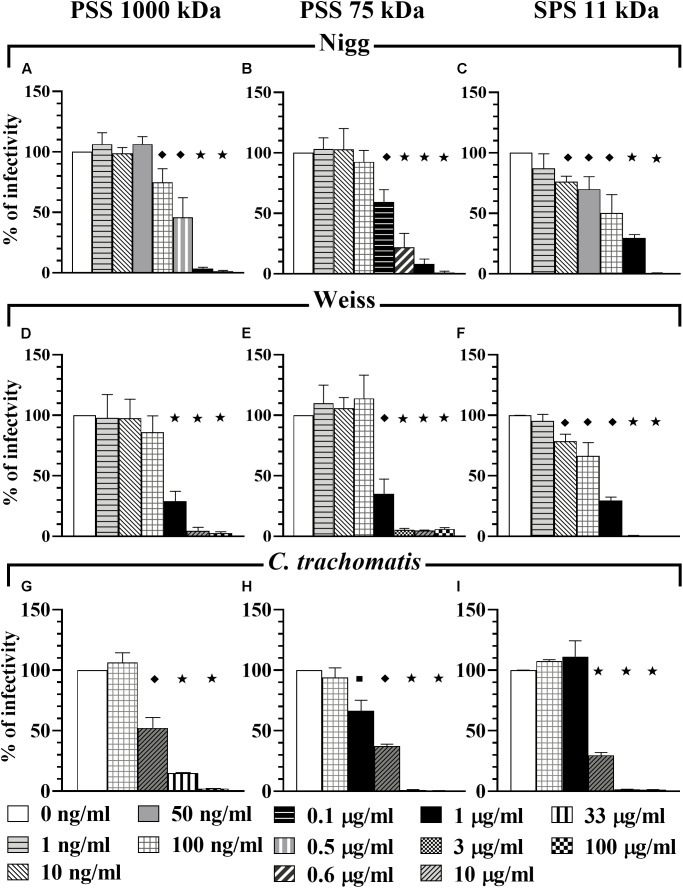
Co-treatment activity of PSS 1000 kDa, PSS 75 kDa, and SPS 11 kDa against *C. muridarum* Nigg and Weiss strains and *C. trachomatis*. *C. muridarum* EBs Nigg **(A–C)**, Weiss strain **(D–F)**, and *C. trachomatis* **(G–I)** were used to infect HeLa cells in the presence of different drug concentrations (co-treatment). Multiplicity of infection (MOI) used in this *in vitro* study was 1. After infection, plates were fixed and IFU were detected by immunofluorescence and counted as indicated in Materials and Methods. The inclusion values of all treatments were normalized to the non-treated control as percentage (% of infectivity). Bars represent the mean per treatment of at least three independent experiments; error bars indicate the standard deviation (SD). Stars (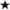) indicate statistical significance when compare to the non-treated control with a *p* < 0.0001, diamonds (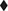) indicate statistical significance with a *p* < 0.008, and squares (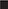) indicate a *p* ≤ 0.03 as calculated by Student’s *t*-test.

**FIGURE 2:**
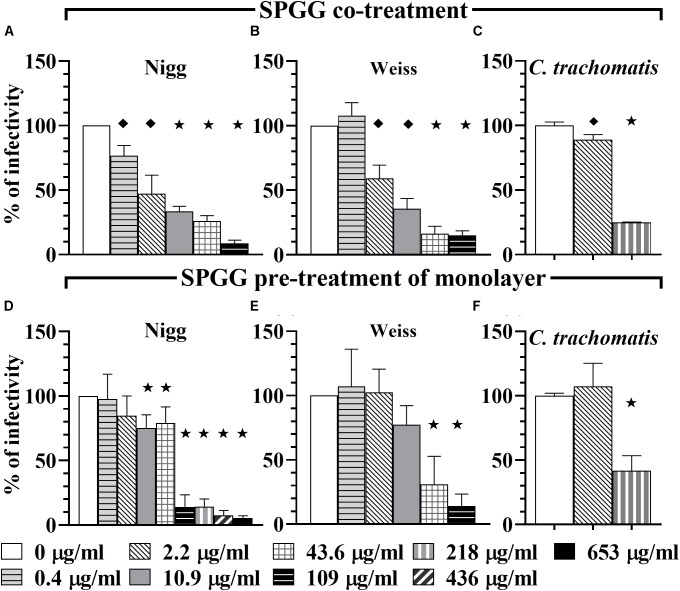
Activity of SPGG against *C. muridarum* (Nigg and Weiss) and *C. trachomatis*. EBs of *C. muridarum* Nigg **(A,D)**, Weiss strain **(B,E)** and *C. trachomatis* **(C,F)** were used to infect HeLa cells in the presence of different drug concentrations (co-treatment, panels **A**–**C**) or monolayers were drug-treated before infection (pre-treatment of monolayers: **D**–**F**). MOI used in this *in vitro* study was 1. After infection, plates were fixed and IFU were detected by immunofluorescence and counted as indicated in Materials and Methods. The inclusion values of all treatments were normalized to the non-treated control as percentage (% of infectivity). Bars represent the mean per treatment of at least three independent experiments, error bars indicate the SD. Stars (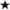) indicate statistical significance when compare to the non-treated control with a *p* < 0.0001, diamonds (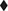 indicate statistical significance with a *p* ≤ 0.007, and squares (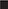) indicate a *p* ≤ 0.01 as calculated by Student’s *t*-test.

To determine the anti-*Chlamydia* activity of the compounds tested in this study, we initially infected HeLa 229 cells with *C. muridarum* strains Nigg or Weiss or *C. trachomatis* in the presence of compounds in a dosage dependent manner (co-treatment). Mock treated cells infected with corresponding *Chlamydia* strain served as a non-treated control (positive control). All four compounds – PSS 1000 kDa (Figures 1A,D,G), PSS 75 kDa (Figures 1B,E,H), SPS 11 kDa (Figures 1C,F,I), and SPGG (Figures 2A–C) – inhibited *Chlamydia* growth. However, the dose-response inhibition curves were different between the tested compounds (Figures 1, 2 and Table 1).

**Table 1:** Effects of the sulfated or sulfonated compounds on infection with *Chlamydia spp.* during co-treatment.

Sulfated or sulfonated compound	*Chlamydia* strain	IC_50_ (μg/ml)	IC_90_ (μg/ml)	IC_50_ 95% CI^#^ (μg/ml)
PSS 1000 kDa	Nigg	0.3	1.4	0.2–0.4
	Weiss	0.6	1.9	0.3–0.8
	*C. trachomatis*	10	42	8.7–12
PSS 75 kDa	Nigg	0.3	0.9	0.3–0.4
	Weiss	0.8	1.4	0.5–0.9
	*C. trachomatis*	3.4	66	1.7–6.7
SPS 11 kDa	Nigg	0.5	8.0	0.3–1.0
	Weiss	0.4	4.5	0.3–0.7
	*C. trachomatis*	7.9	13	5.5–8.8
SPGG	Nigg	1.3	230	0–3.2
	Weiss	2.4	12	1.3–3.7


*^#^CI: confidence interval*.

PSS 1000 kDa, PSS 75 kDa, and SPS 11 kDa inhibited *C. muridarum* Nigg strain with IC_50_ ranging from 0.3 to 0.5 μg/ml while Weiss strain IC_50_ ranged from 0.4 to 0.8 μg/ml (Figure 1 and Table 1). Inhibition of *Chlamydia* Nigg strain at 90% (IC_90_) was achieved at concentrations of 1.4 μg/ml PSS 1000 kDa, 0.9 μg/ml PSS 75 kDa, and 8 μg /ml SPS 11 kDa, while Weiss strain inhibition was at 1.9, 1.4, and 4.5 μg/ml, respectively (Table 1). Inhibition of *C. trachomatis* was less effective, IC_50_ of PSS 1000 kDa was 10 μg/ml, PSS 75 kDa was 3.4 μg/ml, and SPS 11 kDa was 7.9 μg/ml; while IC_90_ ranged from 13 to 66 μg/ml. From all the drugs tested in this study, SPGG is the most effective in blocking *C. muridarum*. The inhibitory activity of SPGG against *C. muridarum* Nigg shows an IC_50_ 1.3 μg/ml while Weiss strain was 2.4 μg/ml (Figures 2A,B and Table 1). *C. trachomatis* infectivity was significantly reduced down to 20% infectivity with 218 μg/ml SPGG (Figure 2C). These results show that all compounds inhibited *Chlamydia* similarly in the micromolar range. However, these results could not distinguish whether the inhibition resulted from binding to chlamydial ligands or host cell receptors.

### Blocking Host Cell *Chlamydia* Receptors

**FIGURE 3:**
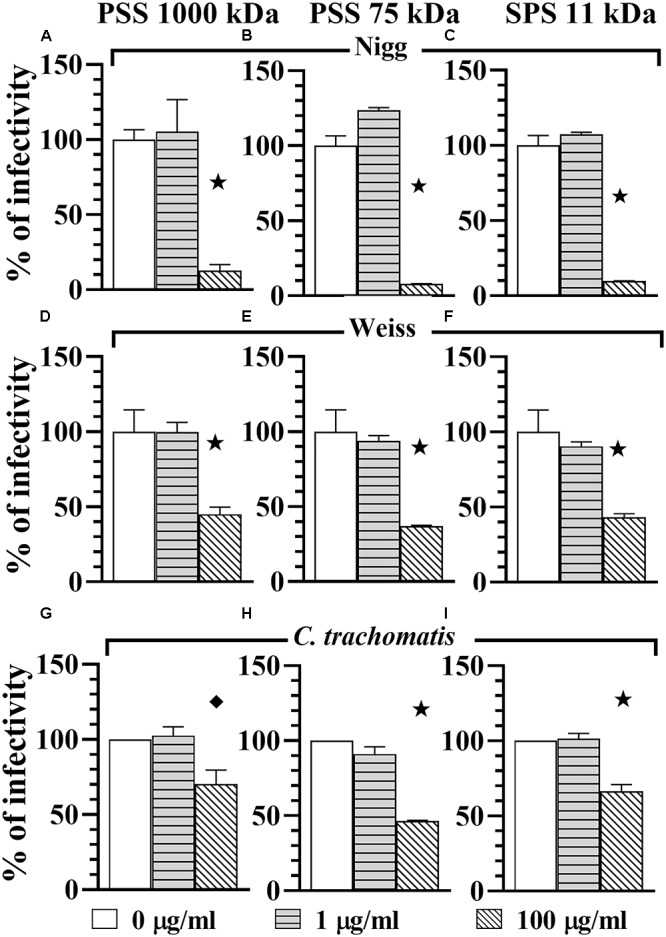
Host-cell blocking activity of PSS 1000 kDa, PSS 75 kDa, and SPS 11 kDa against *C. muridarum* Nigg and Weiss strains and *C. trachomatis*. *Chlamydia* EBs Nigg **(A–C)**, Weiss strain **(D–F)**, and *C. trachomatis* **(G–I)** were used to infect HeLa cells. Monolayers were drug-treated before infection (pre-treatment of monolayers). MOI used in this *in vitro* study was 1. After infection, plates were fixed and IFU were detected by immunofluorescence and counted as indicated in Materials and Methods. The inclusion values of all treatments were normalized to the non-treated control as percentage (% of infectivity). Bars represent the mean per treatment of two independent experiments, error bars indicate the SD. Stars (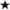) indicate statistical significance when compare to the non-treated control with a *p* < 0.0001, and diamonds (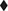) indicate statistical significance with a *p* ≤ 0.007 as calculated by Student’s *t*-test.

In order to determine if PSS 1000 kDa, PSS 75 kDa, SPS 11 kDa and SPGG were able to block attachment or adhesion of *Chlamydia* by interacting with host cells, we pre-treated the HeLa 229 monolayers for 30 min with different concentrations of drugs. The excess of drug was removed by pipetting and the wells were washed once with PBS. Then wells were infected with *Chlamydia* as per standard protocol. Our results show that at 1 μg/ml of PSS 1000 kDa, PSS 75 kDa, SPS 11 kDa, no reduction of *C. muridarum* Nigg (Figures 3A–C), Weiss (Figures 3D–F) or *C. trachomatis* (Figures 3G–I) infection was observed. On the other hand, a relatively higher concentration of 100 μg/ml of all three compounds inhibited *C. muridarum* Nigg to approximately 10% infectivity while Weiss strain was only reduced by ∼60% infectivity (Figures 3A–F). For *C. trachomatis*, inhibition was less effective. Some compounds only maintained some residual activity; PSS 1000 kDa and SPS 11 kDa only inhibited ∼30%, and PSS 75 kDa ∼60% (Figures 3G–I). Pretreatment of the host cell monolayers with SPGG was effective to reduce 90% of infectivity of *C. muridarum* at concentrations of 218 μg/ml (Figures 2D, E), while only 60% inhibition of *C. trachomatis* infectivity was observed at similar concentrations (Figure 2F). These results showed the assessed compounds reduce attachment of *C. muridarum* and *C. trachomatis* to host cell only at 100 μg/ml PSS 1000 kDa, PSS 75 kDa, SPS 11 kDa and 43.6 μg/ml SPGG; and it is less effective to reduce *C. trachomatis* than *C. muridarum*. Therefore, the compounds might be working at the point of attachment blocking interaction of *Chlamydia* with the eukaryotic cell surface receptor but pre-treatment of the host cell is not as effective as when the compounds are present together with chlamydial EBs at the time of infection.

### Blocking *Chlamydia* EBs

**FIGURE 4:**
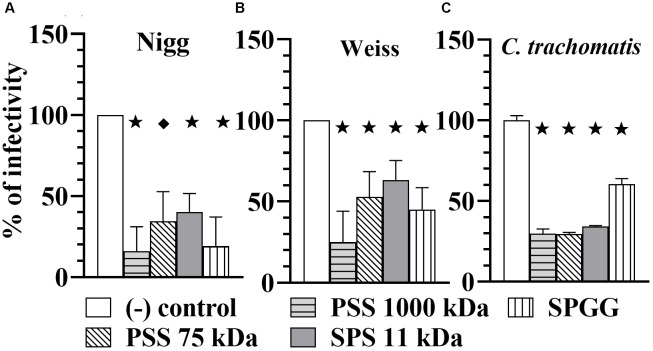
Microbial-blocking activity of PSS 1000 kDa, PSS 75 kDa, SPS 11 kDa and SPGG against *C. muridarum* Nigg **(A)**, Weiss **(B)** strains and *C. trachomatis* **(C)**. *Chlamdyia* EBs were drug-treated with 100 μg/ml of PSS 1000 kDa, PSS 75 kDa, SPS 11 kDa, or 218 μg/ml SPGG before infecting HeLa cells. MOI used in this *in vitro* study was 3. After infection, plates were fixed and IFU were detected by immunofluorescence and counted as indicated in Materials and Methods. The inclusion values of all treatments were normalized to the non-treated control as percentage (% of infectivity). Bars represent the mean per treatment of at least two independent experiments, error bars indicate the SD. Stars (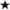) indicate statistical significance when compare to the non-treated control with a *p* < 0.0001, and diamonds (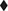 indicate statistical significance with a *p* ≤ 0.007 as calculated by Student’s *t*-test.

To determine whether chlamydial inhibition resulted from binding of the compounds to chlamydial EB ligands, we pre-treated *Chlamydia* EBs for 30 min with 100 μg/ml of sulfated or sulfonated compounds, removed the drug by centrifugation and then used those EBs to challenge host cell monolayers. Our results show that the pre-treatment of EBs reduced the infectivity of the *Chlamydia*. *C. muridarum* Nigg strain was significantly reduced in infectivity by 80% with PSS 1000 kDa, 60% with both PSS 75 kDa and SPS 11 kDa, and 80% with SPGG (Figure 4A). Similarly, *C. muridarum* Weiss infectivity was reduced by 80, 50, 40, and 50%, respectively. *C. trachomatis* had a reduction of 70% infectivity with all polymer agents (PSS 1000 kDa, PSS 75 kDa, and SPS 11 kDa) and 40% with SPGG. These results show that the sulfated compounds interact with both the eukaryotic host cells (Figures 2D–F, 3) and *Chlamydia* EBs (Figure 4) to block or reduce infection. These experiments indicate that the compounds are likely acting at the point of attachment of *Chlamydia* by interacting with both *Chlamydia* cell surface molecules and eukaryotic cell surface receptors.

### Cytotoxicity

To assess the potential cytotoxicity of the compounds for HeLa cells, cell proliferation and viability was determined as the conversion of water soluble MTT to a purple insoluble formazan product. PSS 75 kDa, and SPS 11 kDa did not display any cytotoxicity for HeLa cells at concentrations up to 1000 μg/ml, whereas PSS 1000 kDa reduced cell viability ∼25% at 1000 μg/ml. Similarly, SPGG did not exhibit any cytotoxicity at concentration up to 218 μg/ml (Supplemental Figure S2).

### Efficacy of SPGG in the Mouse Intravaginal Infection Model

Finally, the most effective *in vitro* compound, SPGG against *C. muridarum* infection, was tested to evaluate its efficacy *in vivo*. To further explore its potential as an anti-*Chlamydia* therapy, SPGG was used as a topical pre-treatment in a mouse model. Female Swiss Webster mice were treated intravaginally for 30 min with 50, 500, and 4000 μg/ml of SPGG, SPG buffer only or vehicle only. No vaginal irritation or mouse distress was observed following intravaginal administration of single dose. Microbial burden was monitored by quantification of *Chlamydia* content in intravaginal swabs after *in vitro* infection and immunofluorescence staining.

**FIGURE 5:**
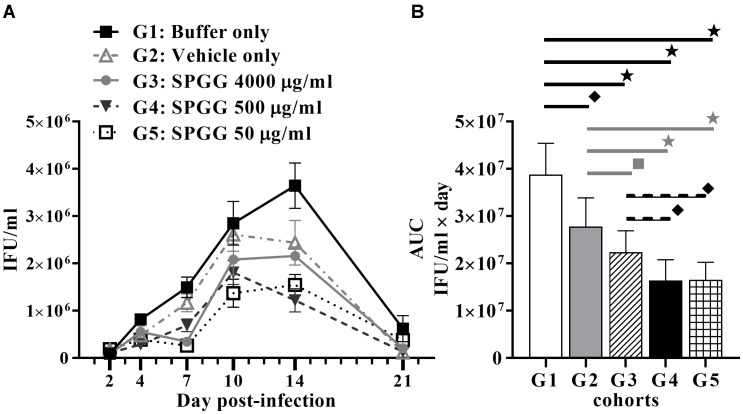
Anti-*Chlamydia* activity of SPGG in mice. **(A)** Swiss Webster Mice were topically treated with SPGG before intravaginal infection with *C. muridarum* Weiss strain. Infection was monitored by intravaginal swabbing on day 2, 4, 7, 10, 14, and 21 post-infection and measuring the *Chlamydia* growth in tissue culture as described in Materials and Methods. Plots represent the mean per treatment of at least ten animals per group, error bars indicate the SD. **(B)** Statistical analysis of the AUC of anti-*Chlamydia* activity of SPGG in mice. AUCs were calculated as indicated in Materials and Methods. G1: Buffer only, G2: Vehicle only, G3: SPGG 4000 μg/ml, G4: SPGG 500 μg/ml, and G5: SPGG 50 μg/ml. Horizontal lines indicate the cohorts compared with statistically significant differences. Stars (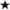) indicate statistical significance of compared cohorts with a *p* ≤ 0.0002, diamonds (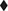 indicate statistical significance with a *p* ≤ 0.0036, and squares (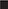) indicate a *p* ≤ 0.021 as calculated by Student’s *t*-test.

In our *in vivo* experiments, all animals of the buffer-only-treated mice became infected and almost all (9/10) animals that received vehicle gel became infected. These data are consistent with no significant protective effect of the buffer or placebo gel. In contrast, while all animals became infected, SPGG gel shows some protective efficacy against *Chlamydia* infection in this model by reducing the microbial burden in 50% or more (Figure 5A). The AUC values of each SPGG-treated cohort (Figures 5B: G3, G4, G5) were compared to the non-treated control value (Figure 5B: G1) using a Student’s *t*-test. This statistical analysis demonstrated that there is significant difference in all drug-treated cohorts compared (*p* ≤ 0.0001). In addition, an ordinary one-way Analysis of Variance (ANOVA) *t*-test was performed comparing all pairs of cohorts resulting in a significant difference of all cohorts (*p* ≤ 0.0001). Even though, we observed a hindering effect of vehicle-only cohort in *Chlamydia* infectivity (Figures 5A,B: G2), when the three SPGG-treated cohorts were compared individually to vehicle-only cohort a significant difference (*p* ≤ 0.0145) was observed using a Student’s *t*-test. In addition, when SPGG-treated cohorts (Figures 5B: G3–G5) were compared between each other, there was significant statically difference (*p* ≤ 0.0145); however, treatment did not display a sigmoidal dose-response curve (Figure 5B). Taken all together, these results demonstrate that a topical gel containing SPGG can reduce microbial burden of *Chlamydia*.

## Discussion

*Chlamydia* is a significant public health problem in the United States and throughout the globe; costing millions of dollars each year. The majority of infected patients, both women and men, are asymptomatic. The lack of treatment causes severe sequelae that prominently include infertility and blindness, making the *Chlamydia sp*. the most common cause of infertility in women and the world’s leading cause of microbial blindness (Centers for Disease Control and Prevention [CDC], 2017). An antimicrobial therapy that prevents chlamydial attachment or reduces infectious burden has the potential to decrease transmissibility or reduce clinical manifestations associated with this infection. However, most therapies available do not prevent C*hlamydia* attachment before infection is established. Thus, there is a need for novel therapeutic strategies to prevent *Chlamydia* infection. The main objective of this study was to compare the effectiveness of *Chlamydia* inhibition of four sulfonated and sulfated agents, PSS 1000 kDa, PSS 75 kDa, SPS 11 kDa, and SPGG as monotherapy against *Chlamydia*, in an attempt to identify a potential compound to prevent or reduce infection. These four compounds, or a derivative thereof, are attractive candidates to treat *Chlamydia* infections because they have been reported to have broad spectrum antimicrobial activity against other pathogens such as *Gardnerella sp.*, *Bacteriodes sp*. (Simoes et al., 2002), herpes virus (Gangji et al., 2018) and HIV (Nakashima et al., 1996).

This study has shown that each polyanionic agent inhibits chlamydial infection in an *in vitro* model. Although each of the agents tested blocked chlamydial infection, SPGG was the most effective in blocking *C. muridarum* in this study. Sulfated polyanions that block *Chlamydia* infection are commonly reported to have multiple sulfate groups that attempt to compete with the sulfated glycosaminoglycan chains present on cell surfaces. Although this is a possible mechanism of inhibition, the precise mode of action of these negatively charged compounds remains to be fully understood. Some reports showed that it depends on the number of charged groups available for interaction with both eukaryotic host cell membrane proteins and microbial surface proteins (Herold et al., 2000; Scordi-Bello et al., 2005). Molecules with several negatively charged groups seem to be more efficient in reducing or blocking infection. On the other hand, the total MW of the compound does not play a substantial role in the inhibition. In this study, our results confirm those findings. The structure of PSS 1000 kDa, PSS 75 kDa, SPS 11 kDa show that each one had a single negatively charged group per monomer and they mainly differ in their MW (Supplemental Figure S1). Our results show that all three compounds had very little difference in IC_50_ values and percent of inhibition in all inhibition studies: co- and pre-treatment of monolayer or *Chlamydia* EBs. In addition, the SPGG structure shows that it has several sulfated groups per molecule and yet only 2.2 kDa MW. It is a highly negatively charged, synthetic small molecule and therefore carries the most promise in this study. Although there was no correlation with MW and efficacy of the compound used, there was a correlation with the degree of sulfation present on the anti-*Chlamydia* agent.

Equally important, SPGG is a highly soluble glucoside that does not have cytotoxic effects on human cervical epithelial tissue culture cells (Supplemental Figure S2). SPGG is also reported to be effective against other STDs such as herpes virus 1 (Gangji et al., 2018) and HIV (Nakashima et al., 1996). Thus, SPGG or similar derivatives deserve further development, more elaborate animal model testing and clinical evaluation. Here, our data illustrate that SPGG inhibits both mouse pathogen *C. muridarum in vitro* and *in vivo* and human pathogen *C. trachomatis*.

Even though promising results are reported here, there are certainly limitations to our study. Our antimicrobial screening was conducted as monotherapy with only two *C. muridarum* strains and one *C. trachomatis* LGV strain. Future studies should evaluate the inhibitory activity of these sulfated agents against other clinically relevant isolates, other *Chlamydia spp.* serovars (e.g., A, E, or D), and in other animal models using a monotherapy or combination therapy. Another potential weakness was the dose of *C. muridarum* used in the mouse. Our group typically uses a *Chlamydia* dose of ∼10^4^ IFU to infect BALB/c or C57BL/6J mice, whereas in this study, a 2 log_10_ higher challenge (10^6^ IFU) was used to infect Swiss Webster mice that required a higher dose to achieve 100% infection. This higher dose may be the reason why, though we observed a reduced infectious burden with doses of SPGG, we did not observe a sigmoid curve or absolute prevention of infection at any concomitantly increasing dose. To further investigate this effect and clarify the inhibitory role on SPGG *in vivo*, new studies should be performed with different mouse strains and several inoculum doses. Also, considering that infectivity of *Chlamydia sp.* serovar LGV strongly depends on a HS-related mechanism; while other urogenital *Chlamydia* serovars (e.g., E) hinge less on HS-like GAGs, a comparison of infection of several *Chlamydia* serovars should be performed to fully understand the inhibitory role of these polyanion compounds. Other animal infection models should also be included in future studies such as the upper urinary tract infection or the trachoma infection model. Finally, we believe the observed hindering effect of the vehicle gel (2% hydroxylcellulose) should be explored in detail to optimize the effect of the polyanion agents, along with the effect of different gel vehicles for drug delivery.

## Conclusion

In conclusion, our results show that SPGG had good inhibitory activity against two *C. muridarum* strains and one *C. trachomatis* LGV strain. This study provides evidence that SPGG topical prophylaxis reduced *Chlamydia* infection and may serve as a potential therapeutic strategy to prevent *Chlamydia* growth of serovars with a GAG-dependent mechanism for infection.

## Ethics statement

This study was carried out in accordance with the recommendations described in the Public Health Service Policy on Humane Care and Use of Laboratory Animals and the Guide for the Care and Use of Laboratory Animals. The MWU animal protocol “Evaluation of sexual transmission of Chlamydia in the mouse” was approved by the MWU Institutional Animal Care and Use Committee (IACUC).

## Author Contributions

KG directed the project, conceived, and designed the experiments with supervision of KR. KG, CT, DR, RDT, DG, and KR performed the experiments. KG, CT, DR, RDT, and DJ contributed in data acquisition. KG analyzed and interpreted the data. VT and UD contributed reagents. KG wrote the manuscript with support from KR. CT proofread the manuscript.

## Conflict of Interest Statement

The authors declare that the research was conducted in the absence of any commercial or financial relationships that could be construed as a potential conflict of interest.

## Supplementary Material

FIGURE S1 Chemical structures of sulfated and sulfonated compounds tested in this study.

FIGURE S2 Cytotoxicity of HeLa cells after 24 h exposure with PSS 1000 kDa, PSS 75 kDa, SPS 11 kDa, and SPGG. MTT assay was performed as indicated in Materials and Methods. OD values were normalized to percent of the non-treated control. Bars represent the mean per treatment of at least two independent experiments, error bars indicate the SD. Stars () indicate statistical significance when compare to the non-treated control with a *p* < 0.0001, and diamonds () indicate statistical significance with a *p* < 0.005 as calculated by Student’s *t*-test.
