# Supplementary figures and images for: A Synthetic, Small, Sulfated Agent Is a Promising Inhibitor of Chlamydia spp. Infection in vivo
Source: Front Microbiol. 2019 Jan 16;9:3269. doi: 10.3389/fmicb.2018.03269 (PMC6343517; doi:10.3389/fmicb.2018.03269)

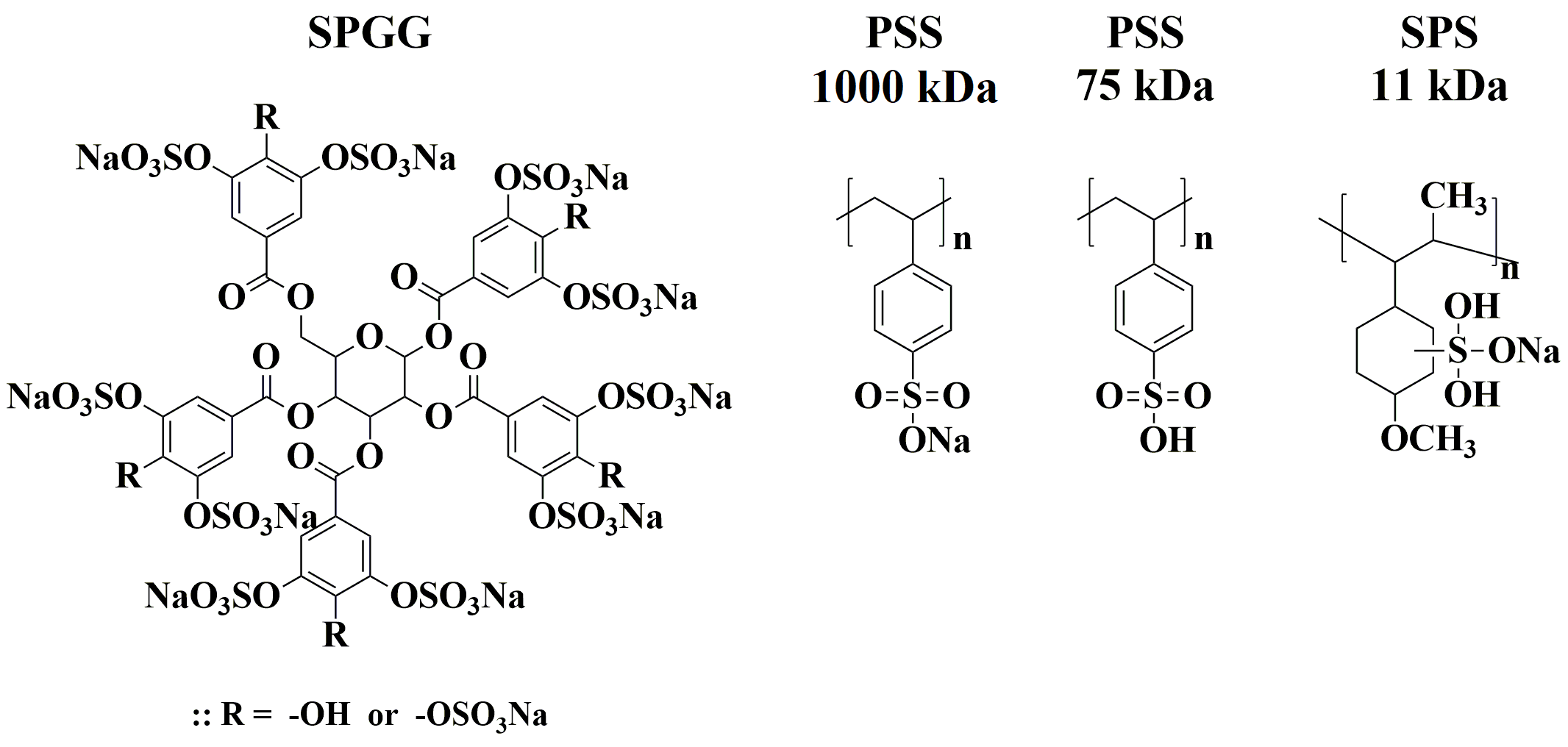

Supplement: FIGURE S1 — Chemical structures of sulfated and sulfonated compounds tested in this study. [file Image_1.TIF]

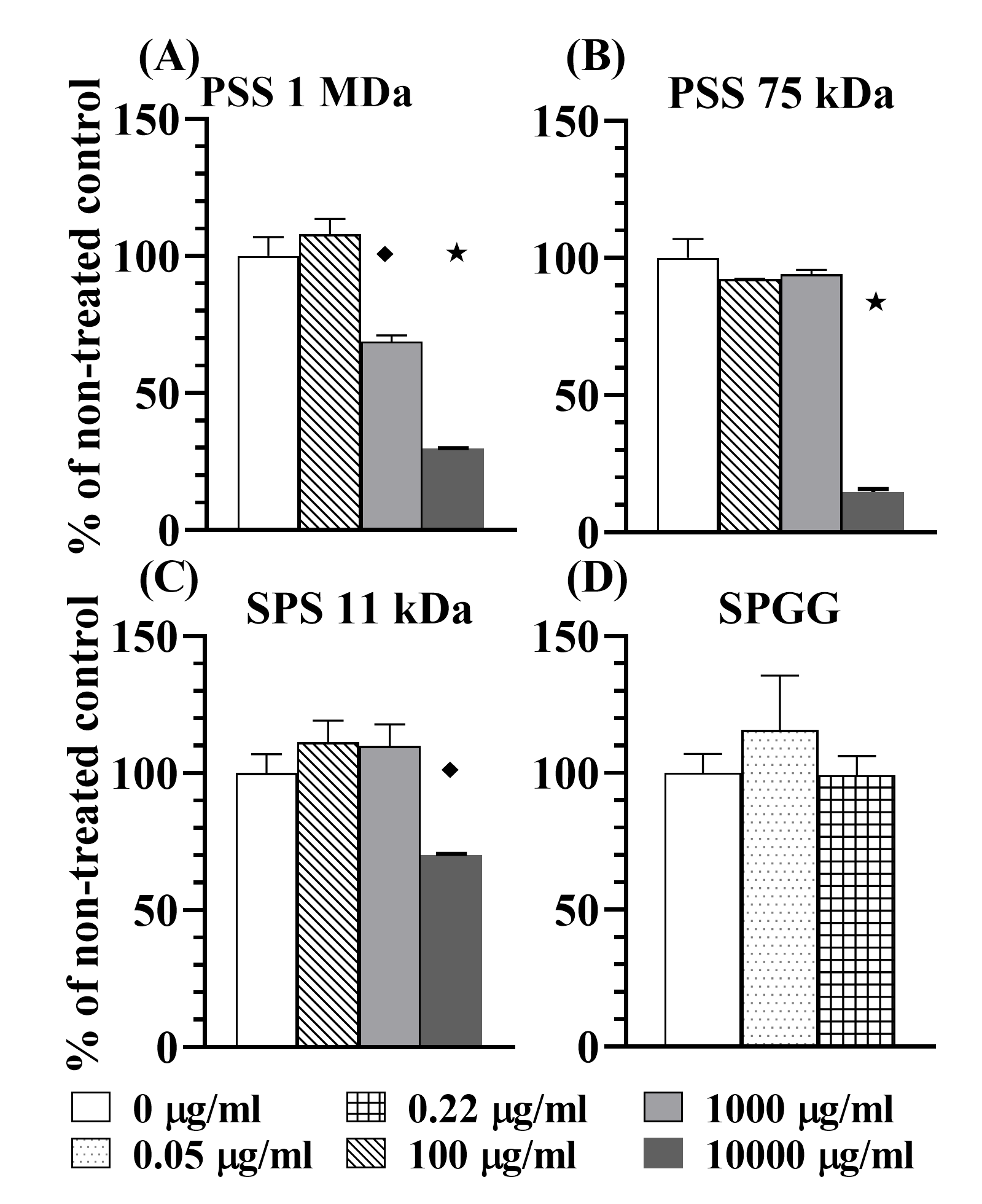

Supplement: FIGURE S2 — Cytotoxicity of HeLa cells after 24 h exposure with PSS 1000 kDa, PSS 75 kDa, SPS 11 kDa, and SPGG. MTT assay was performed as indicated in Materials and Methods. OD values were normalized to percent of the non-treated control. Bars represent the mean per treatment of at least two independent experiments, error bars indicate the SD. Stars () indicate statistical significance when compare to the non-treated control with a p < 0.0001, and diamonds () indicate statistical significance with a p < 0.005 as calculated by Student’s t-test. [file Image_2.TIF]
